# The effect of conditioning regimen intensity on periodontal health in haematopoietic cell transplantation recipients: a 5-year multicentre prospective cohort study

**DOI:** 10.1007/s00784-025-06393-3

**Published:** 2025-06-12

**Authors:** Lucky L.A. van Gennip, Marjolein S. Bulthuis, Renske Z. Thomas, Ewald M. Bronkhorst, Gerjon Hannink, Alexa M.G.A. Laheij, Judith E. Raber-Durlacher, Frederik R. Rozema, Michael T. Brennan, Inger von Bültzingslöwen, Nicole M.A. Blijlevens, Stephanie J.M. van Leeuwen, Marie-Charlotte D.N.J.M. Huysmans

**Affiliations:** 1 Department of Dentistry, Radboud University Medical Center, Nijmegen, The Netherlands; 2 Department of Medical Imaging, Radboud University Medical Center, Nijmegen, The Netherlands; 3 Department of Oral Medicine, Academic Center for Dentistry Amsterdam, University of Amsterdam and VU Amsterdam, Amsterdam, The Netherlands; 4 Department of Oral and Maxillofacial Surgery, Amsterdam UMC, University of Amsterdam, Amsterdam, The Netherlands; 5 Department of Oral Medicine/Oral & Maxillofacial Surgery, Atrium Health Carolinas Medical Center, Charlotte, NC USA; 6 Department of Otolaryngology/Head and Neck Surgery, Wake Forest University School of Medicine, Winston-Salem, NC USA; 7 Department of Oral Microbiology and Immunology, Institute of Odontology, The Sahlgrenska Academy, University of Gothenburg, Gothenburg, Sweden; 8 Department of Hematology, Radboud University Medical Center, Nijmegen, The Netherlands

**Keywords:** Stem cell transplantation, Conditioning regimen intensity, Dentition, Periodontium, Periodontal disease, Longitudinal clinical study

## Abstract

**Objectives:**

To evaluate periodontal health and its association with conditioning intensity over five years post-HCT.

**Materials and methods:**

This multicentre prospective study included 104 patients from two Dutch centres. Probing pocket depth (PPD), bleeding on probing (BOP), and buccal gingival recession (GR) were assessed pre-HCT and at three (*n* = 34), six (*n* = 45), twelve (*n* = 46), eighteen months (*n* = 30), and five years (*n* = 36) post-HCT. Regression models evaluated associations with conditioning intensity and time since HCT.

**Results:**

HCT recipients had a median age of 58 years; 56% were male, and 59% received an allogeneic transplant. At baseline, mean PPD was 2.3 mm (31% had PPD ≥ 6 mm), mean BOP was 23%, and the median number of teeth with GR ≥ 1 mm was nine. Conditioning intensity was not significantly associated with PPD, GR, or BOP over time. Mean PPD decreased slightly at twelve months post-HCT (-0.21 mm (95%CI -0.28, -0.14)) but increased marginally at five years (0.12 mm (95%CI 0.08, 0.16)) compared to baseline. GR increased gradually with 0.13 mm (95%CI 0.07, 0.19) at twelve months, and 0.16 mm (95%CI 0.10, 0.23) at five years. BOP declined at twelve months (-11% (95%CI -15, -8)) but returned to baseline at five years (-1% (95%CI -5, 4)).

**Conclusions:**

Our results suggest that conditioning intensity does not affect long-term periodontal health. Periodontal changes up to five years post-HCT were small.

**Clinical relevance:**

Conditioning intensity may not be a key determinant of post-HCT periodontal health. Post-HCT periodontal deterioration was not found in our study.

**Supplementary Information:**

The online version contains supplementary material available at 10.1007/s00784-025-06393-3.

## Introduction

Haematopoietic cell transplantation (HCT) is widely used in the treatment of both malignant and non-malignant conditions. It involves the transfer of haematopoietic cells either harvested from the patient’s own body (autologous HCT), or from a matched related or unrelated donor (allogeneic HCT). Prior to transplantation, patients undergo a conditioning regimen that includes chemotherapy and may also involve total body irradiation (TBI). The aim of the conditioning regimen is to eradicate cancer and induce immunosuppression to permit donor engraftment [1]. The intensity of the conditioning regimen can vary substantially based on several factors, including type of HCT and the underlying condition of the patient. Myeloablative conditioning (MAC) used to be the conventional conditioning regimen for all patients with leukaemia or lymphoma [2]. Non-myeloablative (NMA) regimens were developed to make transplantation available for patients ineligible for MAC, because of age or the presence of comorbidities [2]. Reduced intensity conditioning (RIC) is an intermediate category of regimens, which does not fit the definition for MAC or NMA. The extent of immunosuppression correlates with the intensity of the conditioning regimen. MAC leads to profound and prolonged immunosuppression, whereas NMA and RIC regimens lead to less profound immunosuppression.

HCT recipients often face a range of oral complications [3]. These complications can affect various oral tissues, including the oral mucosa, salivary glands, the dentition, musculoskeletal tissues, and periodontal tissues. Periodontal inflammation may pose a risk for neutropenic patients as it potentially induces the translocation of bacteria, endotoxins and inflammatory products into the bloodstream, which may result in bacteraemia, fever and sepsis [4]. Therefore, maintaining periodontal health is critical before, during, and after HCT. However, achieving this goal presents considerable challenges. The conditioning regimen elevates the risk of periodontitis exacerbation, and oral pain can hinder effective oral hygiene practices [5, 6]. A guideline on basic oral care for haemato-oncology patients and HCT recipients has been published, and recently a multi-professional working group clarified the controversies on basic oral care in this vulnerable population [7, 8].

In patients with head and neck cancer, studies have shown significant increases in gingival recession at six, twelve and twenty-four months after radiotherapy compared to pre-treatment levels, while mean probing pocket depth did not increase [9, 10]. However, longitudinal studies specifically focusing on periodontal health in HCT patients remain limited [11]. A prospective study by Pattni et al. reported improvements in periodontal health within six months post-HCT, with most of the improvement occurring in the first three months after HCT [12]. Conversely, severe gingival recession and early loss of teeth have been documented as complications in a young HCT patient [13]. Prospective studies with extended follow-ups are essential as sufficient time may be necessary for periodontal conditions to manifest. This multicentre prospective study evaluated periodontal health in HCT recipients and its association with conditioning intensity over a five-year period following transplantation.

## Methods

### Study design

H-OME is a Dutch ancillary study of the multinational, prospective Orastem study on the impact of oral side effects from conditioning therapy before HCT [14]. Adult patients (≥ 18 years old) scheduled to receive either an autologous or allogeneic HCT at Amsterdam UMC (location AMC) or Radboud University Medical Center (Radboudumc) Nijmegen were included. Patients scheduled for allogeneic HCT were eligible regardless of their diagnosis, while those scheduled for autologous HCT were included only if they were diagnosed with multiple myeloma. Exclusion criteria were the inability to give written and dated informed consent, a pre-planned second HCT, insufficient time before HCT to consider study participation, a planned transfer to another hospital shortly after HCT, and being edentulous without implants. The study was registered in the Dutch Trial Register (NL5645), and approval was obtained from the Medical Research Ethical Committee (NL52117.018.15). All patients provided informed consent before participation. Patients in the H-OME study were assessed at baseline (pre-HCT), and at three and twelve months post-HCT. Allogeneic HCT recipients had additional visits at six and eighteen months post-HCT. A five-year follow-up (HOME2 study, Dutch Trial Register: NL9825) was subsequently added. Surviving participants, both autologous and allogeneic HCT recipients, were invited to participate, except for those who had undergone a second HCT. Ethical approval was waived by the ethical committee of the Radboudumc because this research was not subjected to the law governing research involving human subjects (2022–13453). Renewed informed consent was obtained from all patients prior to participation.

### Study outcomes

Primary study outcomes were probing pocket depth (PPD), percentage bleeding on probing (BOP), and gingival recession (GR) over time. Secondary outcomes were tooth extractions and patient-reported gum pain and gum bleeding over time. PPD and BOP were recorded at six sites per tooth (mesiobuccal, midbuccal, distobuccal, mesiolingual, midlingual, and distolingual), while GR was recorded at one site per tooth (midbuccal). PPD was measured in millimeters from the gingival margin to the bottom of the sulcus, and buccal GR was measured from the enamel-cemental junction to the gingival margin. Since mesial and distal surfaces exhibit greater PPD values than corresponding flat surfaces [15], missing PPD values were imputed using the PPD from the same location (mesial, mid, distal) on the opposite side of the tooth (buccal, lingual). PPD measurements were missing in less than 1% of all pocket sites. BOP was scored after probing as either present or absent, and percentage BOP was calculated by dividing the number of sites with BOP by the total number of sites probed. Periodontal examinations were conducted by trained and calibrated dentists. The number of tooth extractions over time was determined by comparing the dental status at baseline with that at subsequent follow-ups. Patients completed the European Organisation for Research and Treatment of Cancer Quality of Life Questionnaire– Oral Health Module (EORTC QLQ-OH15) at each study visit. The EORTC QLQ-OH15 is a 15-item questionnaire to assess oral health-related quality of life in cancer patients [16]. The first two questions—“Have you had pain in your gums?” and “Have you had problems with bleeding gums?“—were used to assess patient-reported gum pain and gum bleeding. Responses were recorded on a 4-point Likert scale, where a higher score indicated worse oral health-related quality of life.

### Conditioning regimen

The conditioning regimen for each patient was determined based on disease- and patient-related factors. Conditioning regimens were categorized into three distinct intensity levels: MAC, RIC or NMA [2].

### Statistical analysis

The PPD data structure was complex, with multiple levels of nested measurements: individuals were assessed at multiple timepoints, multiple teeth were examined per timepoint, and each tooth had measurements at six sites. To simplify this complexity, we calculated the mean PPD for each tooth by averaging the measurements from its six probing sites. HCT recipients with at least one follow-up examination were included in the statistical analysis. To estimate scores for patients who missed certain study visits, multiple imputation by chained equations was employed under the assumption that data was missing at random. We used predictive mean matching to impute 50 datasets, and subsequently pooled the results across all imputed datasets using the Rubin’s rules [17]. PPD, percentage BOP and GR were analysed using linear mixed-effects models. PPD, percentage BOP and GR were used as dependent variables; and time since HCT, conditioning regimen intensity, and a random intercept for individuals (and for teeth in the PPD and GR analyses) as independent variables. The analyses were then adjusted for age at HCT, sex, centre and the presence of comorbidities. The normality of residuals was assessed using residual plots. Regression coefficients with 95% confidence intervals were reported and visualized using dot-and-whisker plots. All statistical analyses were conducted using R (version 4.1.3; R Foundation for Statistical Computing, Vienna, Austria).

## Results

**Fig. 1:**
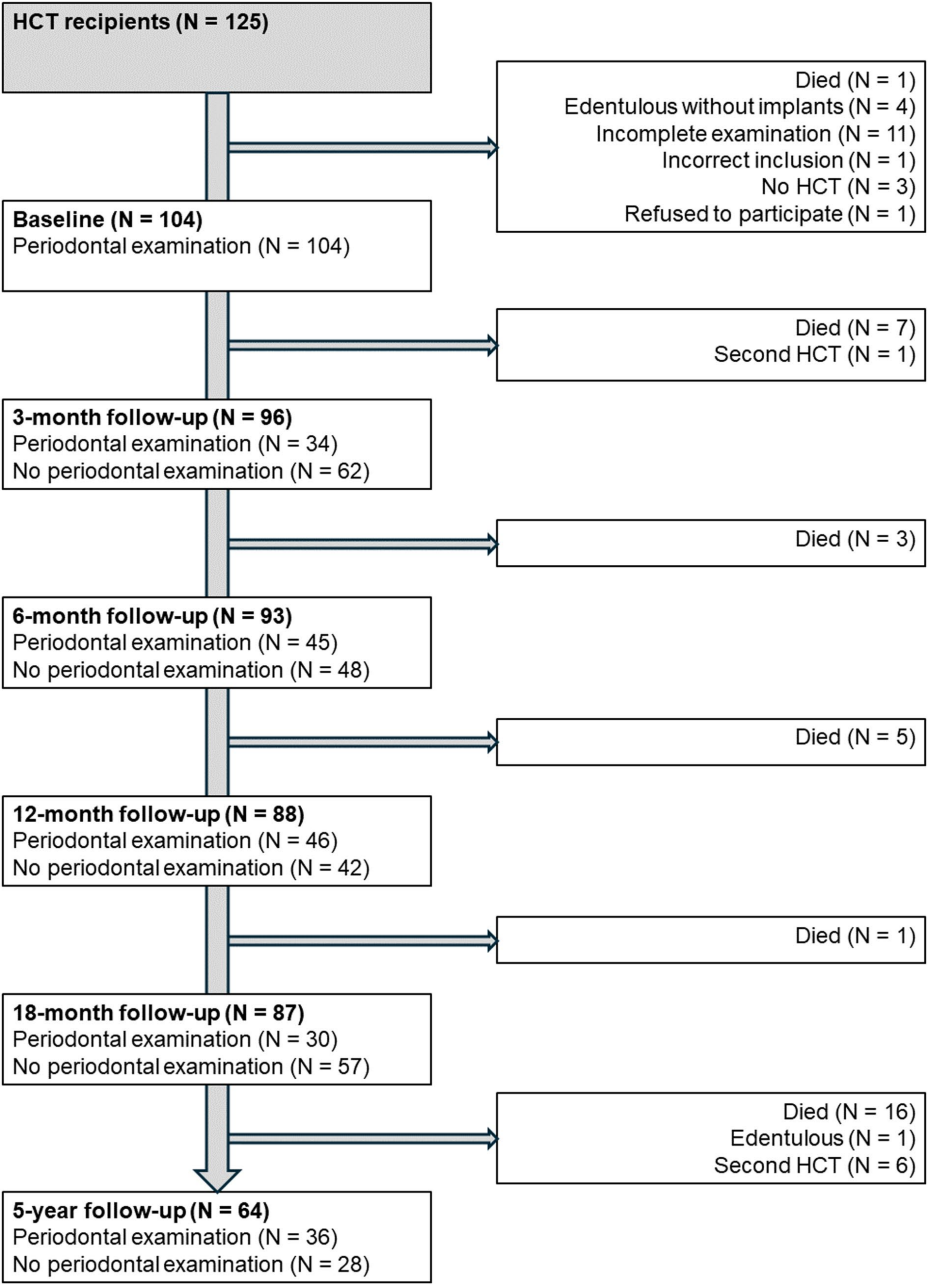
Flowchart of the study

A total of 125 patients scheduled for HCT were enrolled in the study between September 2015 and October 2017. Patient attendance at different follow-ups has been reported previously [18, 19]. At baseline, 104 patients underwent a complete periodontal examination, which was conducted at a median of 35 days (range 3–309 days) prior to HCT. Figure 1 illustrates the number of patients who received a complete periodontal examination at subsequent follow-ups. During the study, 33 patients died, with eight of these deaths occurring before the first follow-up. Eleven patients who did not receive a complete periodontal examination at baseline were excluded from this study. Table 1 presents the baseline characteristics of the total study population and of the subgroups with complete periodontal examinations at three months, twelve months, and five years post-HCT. The majority of patients in the subgroups who underwent complete periodontal examinations at three and twelve months post-HCT received allogeneic transplants and were treated in Nijmegen.

**Table 1:** Baseline characteristics of the total study population (*N* = 104) and of the subgroups with complete periodontal examinations at three months post-HCT (*N* = 34), twelve months post-HCT (*N* = 46), and five years post-HCT (*N* = 36)

	Baseline (*N* = 104)	3-month follow-up (*N* = 34)	12-month follow-up (*N* = 46)	5-year follow-up (*N* = 36)
Age in years, median [IQR]	58 [52–63]	55 [47–63]	55 [47–63]	56 [52–63]
Sex				
- Male - Female	58 (56%) 46 (44%)	18 (53%) 16 (47%)	24 (52%) 22 (48%)	23 (64%) 13 (36%)
Diagnosis				
- AML - ALL - Lymphoma	27 (26%) 4 (4%) 7 (7%)	13 (38%) 4 (12%) 5 (15%)	16 (35%) 2 (4%) 7 (15%)	5 (14%) 2 (6%) 6 (17%)
- CLL	3 (3%)			
- MDS - CML	8 (8%) 2 (2%)	3 (9%) 2 (6%)	5 (11%) 2 (4%)	2 (6%) 1 (3%)
- MF	3 (3%)		2 (4%)	1 (3%)
- SAA - Myeloma	2 (2%) 45 (43%)	2 (6%) 5 (15%)	2 (4%) 7 (15%)	1 (3%) 16 (44%)
- Other	3 (3%)		3 (7%)	2 (6%)
Type of HCT				
- Autologous - Allogeneic	43 (41%) 61 (59%)	3 (9%) 31 (91%)	5 (11%) 41 (89%)	15 (42%) 21 (58%)
Conditioning				
- NMA - RIC - MAC	27 (26%) 22 (21%) 55 (53%)	14 (41%) 9 (26%) 11 (32%)	15 (33%) 18 (39%) 13 (28%)	7 (19%) 9 (25%) 20 (56%)
TBI	42 (40%)	23 (68%)	25 (52%)	12 (33%)
Centre				
- Amsterdam - Nijmegen	37 (36%) 67 (64%)	3 (9%) 31 (91%)	5 (11%) 41 (89%)	9 (25%) 27 (75%)
Number of teeth, median [IQR]	26 [24–28]	27 [23–28]	27 [24–28]	27 [25–28]
Comorbidity	33 (32%)	5 (15%)	12 (26%)	11 (31%)
Smoking				
- Non-smoker - Former smoker - Daily	45 (43%) 52 (50%) 7 (7%)	16 (47%) 17 (50%) 1 (3%)	19 (41%) 25 (54%) 2 (4%)	18 (50%) 17 (47%) 1 (3%)
Alcohol consumption				
- Non-drinker - Former drinker - Not daily - Daily	12 (12%) 43 (41%) 40 (38%) 9 (9%)	3 (9%) 12 (35%) 16 (47%) 3 (9%)	7 (15%) 20 (43%) 17 (37%) 2 (4%)	4 (11%) 13 (36%) 18 (50%) 1 (3%)
Tooth brushing				
- Once per day - Twice per day - Three or more times per day	23 (22%) 68 (65%) 13 (13%)	5 (15%) 25 (74%) 4 (12%)	9 (20%) 32 (70%) 5 (11%)	7 (19%) 25 (69%) 4 (11%)
Interdental cleaning				
- Less than once per week - Less than once per day– once per week - Once per day - Twice per day - Three or more times per day *Missing*	35 (34%) 16 (15%) 39 (38%) 7 (7%) 6 (6%) *1 (1%)*	12 (35%) 4 (12%) 13 (38%) 2 (6%) 2 (6%) *1 (3%)*	15 (33%) 7 (15%) 19 (41%) 3 (7%) 2 (4%)	12 (33%) 9 (25%) 11 (31%) 2 (6%) 2 (6%)
Dental visit				
- Never - Only for acute problems - Routinely	3 (3%) 8 (8%) 93 (89%)	1 (3%) 4 (12%) 29 (85%)	2 (4%) 4 (9%) 40 (87%)	1 (3%) 1 (3%) 34 (94%)

Values are N (%) unless otherwise stated. Abbreviations: IQR = interquartile range, AML = acute myeloid leukaemia, ALL = acute lymphocytic leukaemia, CLL = chronic lymphocytic leukaemia, MDS = myelodysplastic syndrome, CML = Chronic myeloid leukaemia, MF = Myelofibrosis, SAA = severe aplastic anaemia, HCT = haematopoietic cell transplantation, NMA = non-myeloablative, RIC = reduced intensity, MAC = myeloablative, TBI = total body irradiation

### Probing pocket depth

**Fig. 2:**
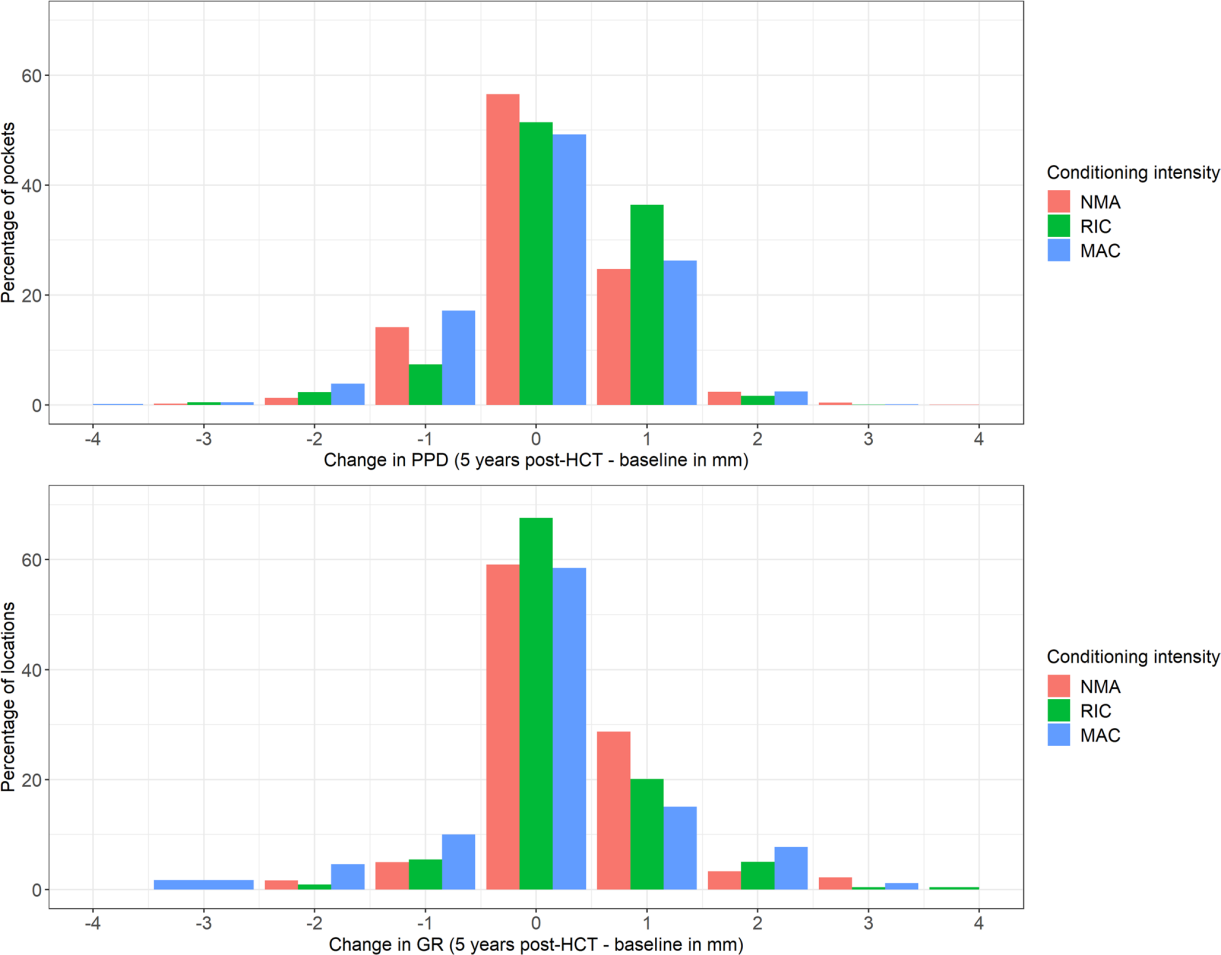
Changes in individual pocket site measurements and buccal gingival recession at five years post-HCT compared to baseline (*N* = 36). The x-axes represent changes in probing pocket depth (PPD) and gingival recession (GR) in millimetres, while the y-axes indicate the percentage of sites. The upper graph shows changes in 5,514 pocket sites, and the lower graph shows changes in 919 buccal locations. Abbreviations: PPD = probing pocket depth; GR = gingival recession; NMA = non-myeloablative; RIC = reduced intensity conditioning; MAC = myeloablative conditioning

At baseline, 104 patients underwent a complete periodontal examination. A total of 123 teeth in 32 patients (31%) had at least one pocket site with a PPD of ≥ 6 mm. Four of the 32 HCT recipients with deep periodontal pockets (PPD ≥ 6 mm) did not attend any follow-up. In the remaining 28 patients, only 16 teeth were extracted over the observation period. At three months post-HCT, 18 teeth in seven patients (21%) had a PPD of ≥ 6 mm, which further decreased by 12 months to nine teeth in six patients (13%). At five years post-HCT, 16 teeth in six patients (17%) had at least one pocket site with a PPD of ≥ 6 mm. Mean PPD was 2.3 mm (SD 0.9, range 1–12) at baseline, 2.3 mm (SD 0.9, range 1–8) at three months, 2.2 mm (SD 0.9, range 1–9) at six months, 2.2 mm (SD 0.8, range 1–8) at twelve months, 2.1 mm (SD 0.8, range 1–7) at eighteen months, and 2.4 mm (SD 0.9, range 1–10) at five years. Figure 2 illustrates individual pocket site measurements at five years of follow-up compared to baseline values, categorized by conditioning intensity. More than half of the pocket sites remained stable throughout the observation period.

### Gingival recession

At baseline, HCT recipients had a median of nine teeth (IQR 2–14) with a buccal gingival margin level at least one millimetre below the enamel-cemental junction. At three months post-HCT, this increased to a median of eleven teeth (IQR 2–16), which remained stable at 12 months (median eleven teeth, IQR 2–16). By five years, the median number of teeth with a buccal gingival margin level at least one millimetre below the enamel-cemental junction increased to thirteen teeth (IQR 4–18). Mean buccal GR was 0.7 mm (SD 1.1, range 0–6) at baseline, 0.8 mm (SD 1.1, range 0–7) at three months, 0.9 mm (SD 1.2, range 0–5) at six months, 0.8 mm (SD 1.2, range 0–6) at twelve months, 1.0 mm (SD 1.3, range 0–7) at eighteen months, and 0.7 mm (SD 1.0, range 0–6) at five years. At the three-month follow-up, the gingival margin level remained unchanged in 620 out of 795 buccal locations (78%). At the twelve-month follow-up, the gingival margin level did not change in 811 out of 1,111 buccal locations (73%). At the five-year follow-up, 558 out of 919 buccal locations (61%) showed no change in gingival margin level compared to baseline (Fig. 2).

**Fig. 3:**
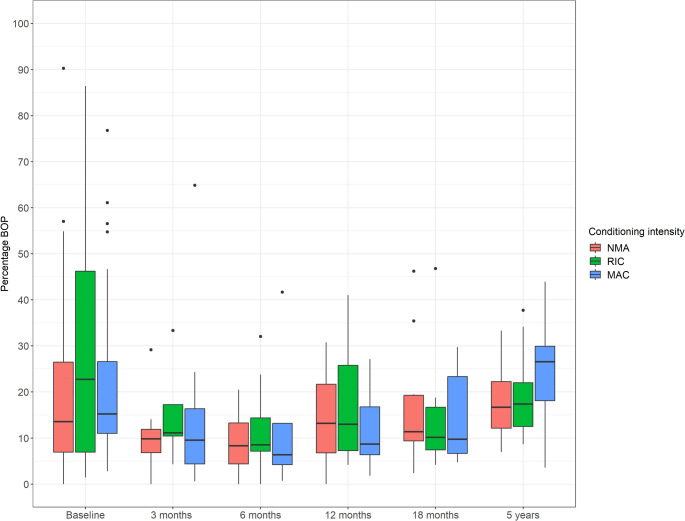
Percentage bleeding on probing at various timepoints: baseline (*N* = 104), three-month follow-up (*N* = 34), six-month follow-up (*N* = 45), twelve-month follow-up (*N* = 46), eighteen-month follow-up (*N* = 30), and five-year follow-up (*N* = 36). Abbreviations: BOP = bleeding on probing; NMA = non-myeloablative; RIC = reduced intensity conditioning; MAC = myeloablative conditioning

**Fig. 4:**
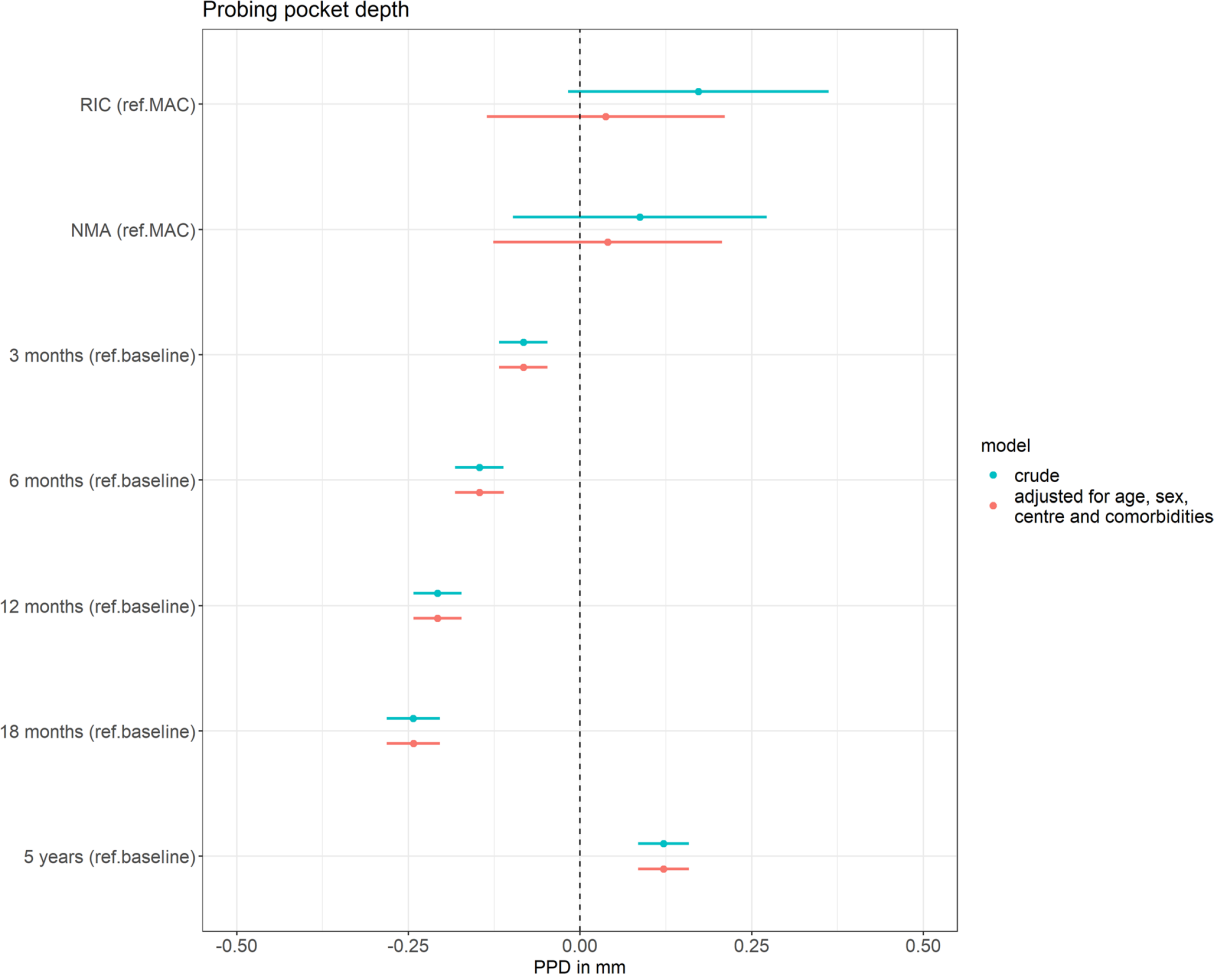
Results of the linear mixed-effects models evaluating two associations: (1) the association between conditioning intensity (reference: MAC) and mean probing pocket depth (PPD) per tooth, adjusted for time since HCT, and (2) the association between time since HCT (reference: baseline) and mean PPD per tooth, adjusted for conditioning intensity. The model is additionally adjusted for age, sex, centre and the presence of comorbidities. Abbreviations: PPD = probing pocket depth; NMA = non-myeloablative; RIC = reduced intensity conditioning; MAC = myeloablative conditioning

### Bleeding on probing

At baseline, HCT recipients had a mean precentage BOP of 23.3% (SD 20.4). At three months post-HCT, the mean percentage BOP decreased to 12.5% (SD 11.8). At 12 months, mean percentage BOP was 14.1% (SD 10.1). By five years, the mean percentage BOP returned to 22.0% (SD 10.8). Figure 3 illustrates percentage BOP over the complete observation period categorized by conditioning intensity.

### Tooth extraction

HCT recipients prepared with NMA conditioning had a mean annual extraction rate of 0.33 (SD 0.87). Those conditioned with RIC had a mean annual extraction rate of 0.18 (SD 0.61), while recipients conditioned with MAC showed a mean rate of 0.22 (SD 0.48). A total of 39 teeth were extracted in 22 patients during the observation period. More than three-quarters of patients did not undergo any tooth extractions during the observation period.

### Patient-reported gum pain and gum bleeding

Table S1 presents the mean scores for patient-reported gum pain and gum bleeding on a 1 to 4 Likert scale, where higher scores indicate a lower quality of life. At baseline, HCT recipients reported a mean gum pain of 1.2 (SD 0.5), which remained stable at three months post-HCT (mean 1.2, SD 0.4). At twelve months, mean gum pain increased to 1.3 (SD 0.6) and remaind at 1.3 (SD 0.4) by five years post-HCT. The proportion of patients reporting no gum pain (answer ‘not at all’) was 83% at baseline, fluctuating between 71% and 83% throughout follow-up. For gum bleeding, the mean score at baseline was 1.3 (SD 0.6). This score decreased to 1.1 (SD 0.3) at three months post-HCT, but increased again to 1.3 (SD 0.5) by twelve months. At five years, the mean gum bleeding score was 1.2 (SD 0.4). The proportion of patients reporting no gum bleeding (answer ‘not at all’) was 79% at baseline, fluctuating between 71% and 88% throughout follow-up.

### Association with conditioning intensity and time since HCT

A total of 70 patients with a baseline periodontal examination and at least one follow-up examination were included in the statistical analysis. Multiple imputation was used to estimate missing data for patients who did not undergo a periodontal examination at certain study visits.

#### Probing pocket depth

The mean PPD for each tooth was calculated by averaging measurements from the six probing sites. Figure 4 presents the results of the linear mixed-effects models evaluating two associations: (1) the association between conditioning intensity and mean PPD per tooth, adjusted for time since HCT and additional confounders, and (2) the association between time since HCT and mean PPD per tooth, adjusted for conditioning intensity and additional confounders. After adjusting for time since HCT, age, sex, centre, and the presence of comorbidities, patients conditioned with RIC and NMA showed a nearly identical mean PPD compared to those conditioned with MAC (effect RIC: 0.04 mm (95%CI -0.14, 0.21); effect NMA: 0.04 mm (95%CI -0.13, 0.21)). Throughout the follow-up period, changes in mean PPD were minimal, with slight reductions compared to baseline at three months (-0.08 mm (95%CI -0.12, -0.05)), six months (-0.15 mm (95%CI -0.18, -0.11)), twelve months (-0.21 mm (95%CI -0.24, -0.17)), and eighteen months (-0.24 mm (95%CI -0.28,-0.20)) post-HCT. At the five-year follow-up, pockets were marginally deeper, with a mean difference of only 0.12 mm compared to baseline (95%CI 0.08, 0.16).

#### Gingival recession

**Fig. 5:**
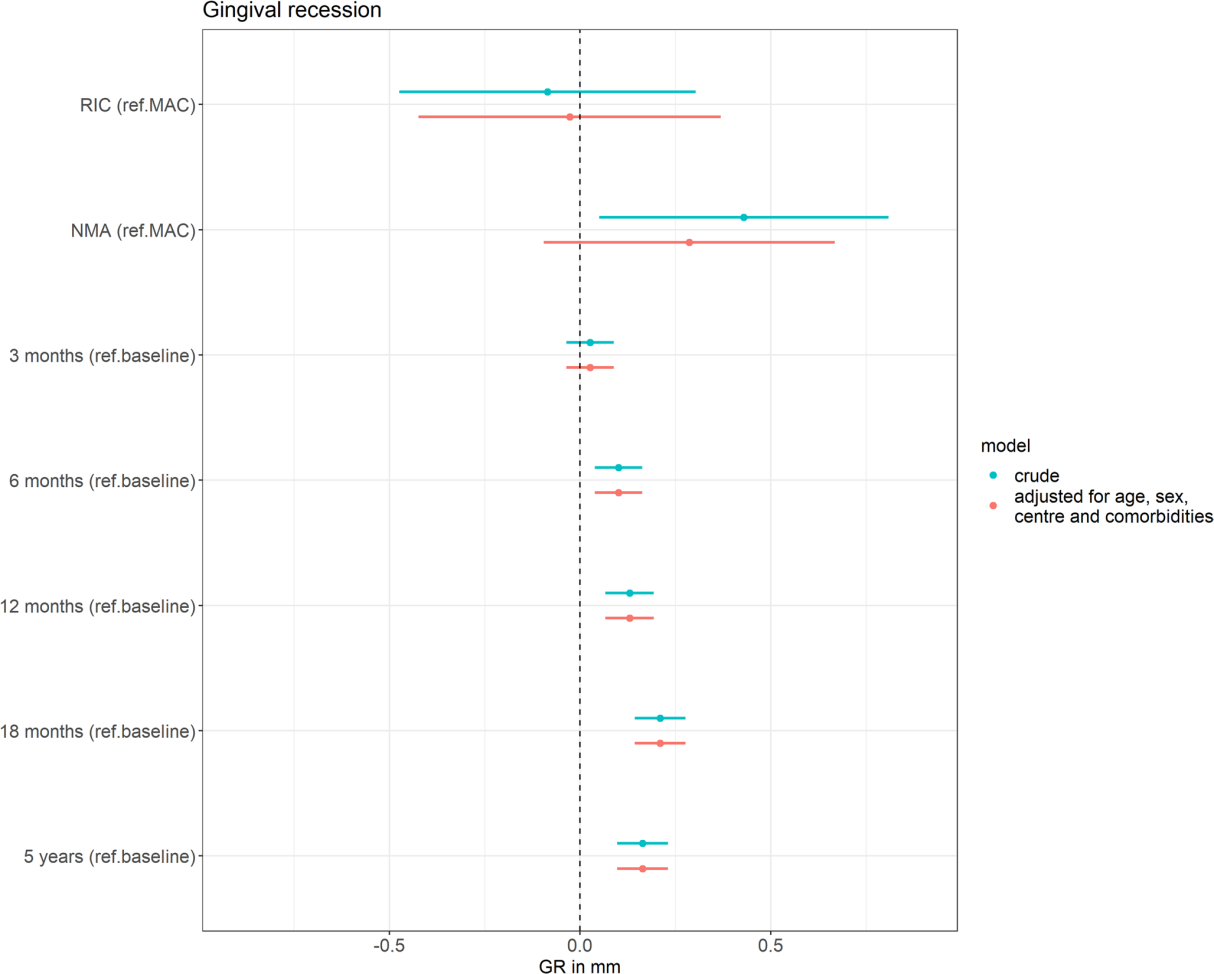
Results of the linear mixed-effects models evaluating two associations: (1) the association between conditioning intensity (reference: MAC) and buccal gingival recession (GR), adjusted for time since HCT, and (2) the association between time since HCT (reference: baseline) and buccal GR, adjusted for conditioning intensity. The model is additionally adjusted for age, sex, centre and the presence of comorbidities. Abbreviations: NMA = non-myeloablative; RIC = reduced intensity conditioning; MAC = myeloablative conditioning

Figure 5 presents the results of the linear mixed-effects models evaluating two associations: (1) the association between conditioning intensity and buccal GR, adjusted for time since HCT and additional confounders, and (2) the association between time since HCT and buccal GR, adjusted for conditioning intensity and additional confounders. After adjusting for time since HCT, age, sex, centre and the presence of comorbidities, patients conditioned with RIC had a mean gingival margin level nearly identical to those conditioned with MAC (-0.03 mm (95%CI -0.42, 0.37)). Patients conditioned with NMA had a mean gingival margin level that was only 0.29 mm lower (95%CI -0.10, 0.67) than those conditioned with MAC. Throughout the follow-up period, GR slightly increased compared to baseline, with differences of 0.03 mm (95%CI -0.04, 0.09) at three months, 0.10 mm (95%CI 0.04, 0.16) at six months, 0.13 mm (95%CI 0.07, 0.19) at twelve months, 0.21 mm (95%CI 0.14, 0.28) at eighteen months post-HCT, and 0.16 mm at five years (95%CI 0.10, 0.23).

#### Bleeding on probing

**Fig. 6:**
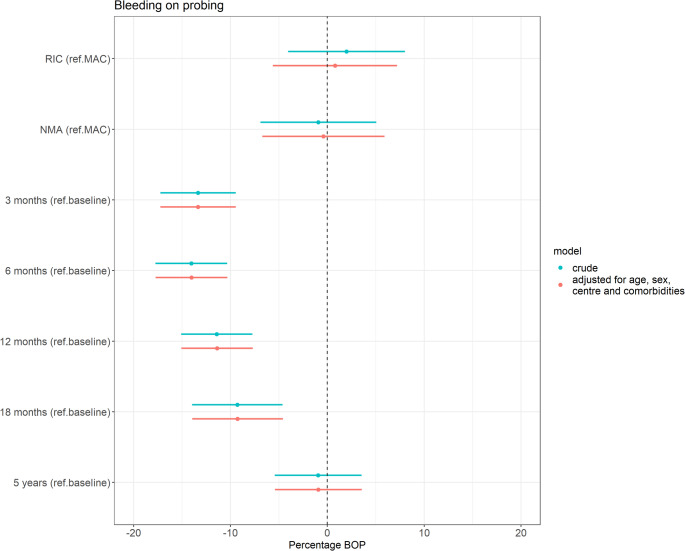
Results of the linear mixed-effects models evaluating two associations: (1) the association between conditioning intensity (reference: MAC) and percentage bleeding on probing (BOP), adjusted for time since HCT, and (2) the association between time since HCT (reference: baseline) and percentage BOP, adjusted for conditioning intensity. The model is additionally adjusted for age, sex, centre and the presence of comorbidities. Abbreviations: BOP = bleeding on probing; NMA = non-myeloablative; RIC = reduced intensity conditioning; MAC = myeloablative conditioning

Figure 6 presents the results of the linear mixed-effects models evaluating two associations: (1) the association between conditioning intensity and percentage BOP, adjusted for time since HCT and additional confounders, and (2) the association between time since HCT and percentage BOP, adjusted for conditioning intensity and additional confounders. After adjusting for time since HCT, age, sex, centre, and the presence of comorbidities, patients conditioned with RIC and NMA had nearly identical mean percentage BOP compared to those conditioned with MAC, with differences of 0.8% (95%CI -5.6%, 7.2%) and − 0.4% (95%CI -6.7%, 5.9%), respectively. Throughout the follow-up period, mean BOP remained slightly lower than baseline levels, with reductions of -13.3% (95%CI -17.2%, -9.5%) at three months, -14.0% (95%CI -17.7%, -10.3%) at six months, -11.4% (95%CI -15.1%, -7.7%) at twelve months, and − 9.3% (95%CI -13.9%, -4.6%) at eighteen months post-HCT. By the five-year follow-up, mean BOP returned to baseline levels (-0.9% (95%CI -5.4%, 3.6%)).

## Discussion

This study evaluated periodontal health in HCT recipients over a five-year period following transplantation, focusing on PPD, BOP, and GR, and its association with conditioning intensity. Conditioning intensity was not significantly associated with these periodontal parameters over time. However, statistically significant temporal variations in periodontal parameters were observed. Mean PPD statistically significantly improved up to eighteen months post-HCT, but showed a slight deterioration at the five-year follow-up compared to baseline. BOP percentages followed a similar trend, with statistically significant reductions up to eighteen months post-HCT, returning to baseline levels at five years post-HCT. GR statistically significantly increased compared to baseline from six months post-HCT onwards. While these changes in periodontal parameters over time were statistically significant, they were generally small in magnitude and not clinically relevant.

Our study found temporal variations in periodontal parameters post-HCT, with minimal initial improvements followed by a return to baseline levels over the five-year period. It is important to note that periodontal health may have already been compromised during the baseline examination due to the underlying illness and accompanying treatments. Previous research compared periodontal health of allogeneic HCT recipients after a mean period of four years post-transplant with that of healthy controls, and showed that survivors had significantly worse periodontal health [20]. While our study did not specifically focus on periodontal treatment pre-HCT, Gürgan et al. reported significant improvements in periodontal status after intensive periodontal treatment prior to HCT, which could be maintained post-HCT [21]. This suggests that targeted periodontal interventions may be beneficial for this patient population. In our study, pre-HCT dental care was provided when necessary and possible, including extractions, endodontic treatments, restorations, supra- and/or subgingival cleaning and oral hygiene instructions. We found that BOP percentages decreased statistically significantly up to eighteen months post-HCT before returning to baseline levels at five years. This aligns with previous research showing significant improvements in gingival index over time, with 63% of the patients demonstrating an improvement in their gingival score from baseline to six months post-HCT [12]. Gingival health may improve initially but may not maintain long-term gains. Pattni et al. also reported improvements in clinical attachment level within six months post-HCT, with most of the improvement occurring in the first three months after HCT, which aligns with the PPD findings in our study [12].

The increased attention on oral health before HCT may have contributed to improved oral hygiene. As periodontal health is closely related to the presence of plaque, enhanced brushing by HCT recipients likely induced improvements in periodontal health, reflected in the decrease of mean PPD and percentage BOP up to eighteen months post-HCT [22]. HCT recipients were referred back to their own dentists after twelve or eighteen months post-HCT. Although Dutch patients are used to regular dental visits, there may be less emphasis on oral health from twelve or eighteen months post-HCT onwards. This could potentially explain PPD and BOP returning to baseline levels during this period. Additionally, our study cohort was five years older at the latest follow-up. Since age is a known risk factor for periodontal disease, it may partly explain the observed trends, particularly the slight increase in GR from baseline [23]. HCT recipients receive immunosuppressive, antiviral and antimicrobial medications, each of which may influence periodontal health. The initiation and progression of periodontal disease are determined by a complex interplay between pathogenic microorganisms in the biofilm and the host inflammatory response [24]. The use of immunosuppressive medications in allogeneic HCT recipients may have contributed to the improvements in periodontal health. It has been suggested that active herpesviruses and specific pathogenic bacteria constitute the main aetiology of severe periodontitis [25]. The use of antivirals in these patients may also have positively influenced periodontal health. Furthermore, the conditioning regimen and frequent use of antibiotics may alter the oral microbiome. It is well established that systemic adjunctive antimicrobials during the active phase of periodontal treatment lead to an additional full-mouth PPD reduction [26, 27]. However, previous research in the same population indicated that although the diversity and composition of the oral microbiome significantly change after HCT, they return to pre-treatment levels from three months post-HCT onwards [28, 29]. Noteworthy, there was no dominance of bacteria typically associated with periodontitis [28, 29].

This study had several limitations that should be considered when interpreting the results. Firstly, to simplify the analysis, the mean PPD per tooth was calculated by averaging measurements from six probing sites. While averaging before analysis is not ideal, a comparison with an alternative multilevel approach showed no impact on the results. Secondly, measuring GR presented significant challenges. The enamel-cemental junction, which serves as a reference point, is often difficult to visualize and cervical restorations and non-carious cervical lesions may obscure this junction. Future studies could benefit from employing 3D scanning technology for more precise and reproducible assessments. Furthermore, evaluating GR at interproximal sites proved even more complex. Consequently, gingival margin levels were only assessed at the midbuccal and midlingual sites, which limited our ability to calculate the clinical attachment level. To capture minor changes in GR more effectively, future research would benefit from including additional measurements, such as recession width, width of keratinized gingiva, gingival thickness, and the percentage of root coverage. Thirdly, multiple dentists conducted the measurements throughout the study. Although all dentists underwent training and calibration to standardize their assessment techniques, the involvement of multiple examiners could have influenced the results. Fourthly, a large proportion of patients at each study visit did not undergo a complete periodontal examination. We assumed that the data were missing at random, meaning that the likelihood of missing data was related to observed variables, such as type of HCT and centre, but not to the unobserved data. Multiple imputation was performed to avoid bias due to missing data during analysis. Fifthly, oral chronic Graft-versus-Host Disease (cGvHD) has been associated with severe GR in the literature [13]. Unfortunately, due to the low incidence of oral cGvHD in our study cohort, we were unable to analyse this association. Lastly, we had limited information on smoking habits over the study period. Smoking habits were documented at baseline and again at the five-year follow-up visit. Although detailed smoking data over time were unavailable, we assume the number of smokers remained low throughout the study period.

In conclusion, this study provides valuable insights into the long-term periodontal health of HCT recipients. Our findings indicate that the intensity of the conditioning regimen may not be a key determinant of post-HCT periodontal health. The minimal changes observed in periodontal health, even long-term after HCT, alleviate concerns regarding deterioration of the periodontium. This study enhances our understanding of periodontal health trajectories in HCT patients and may contribute to oral health management strategies for this population.

## Electronic supplementary material

Below is the link to the electronic supplementary material.

Supplementary Material 1

## Data Availability

The data that support the findings of this study are available under request from the first author.
