# Supplementary material for: The effect of conditioning regimen intensity on periodontal health in haematopoietic cell transplantation recipients: a 5-year multicentre prospective cohort study
Source: Clin Oral Investig. 2025 Jun 12;29(7):338. doi: 10.1007/s00784-025-06393-3 (PMC12159097; doi:10.1007/s00784-025-06393-3)
Supplement: Supplementary file 1 — Supplementary Material 1 [file 784_2025_6393_MOESM1_ESM.docx]

**The effect of conditioning regimen intensity on periodontal health in haematopoietic cell transplantation recipients: a 5-year multicentre prospective cohort study**

Lucky L.A. van Gennip^1^, Marjolein S. Bulthuis^1^, Renske Z. Thomas^1^, Ewald M. Bronkhorst^1^, G. Hannink^2^, Alexa M.G.A. Laheij^3,4^, Judith E. Raber-Durlacher^3,4^, Frederik R. Rozema^3,4^, Michael T. Brennan^5,6^, Inger von Bültzingslöwen^7^, Nicole M.A. Blijlevens^8^, Stephanie J.M. van Leeuwen^1^, Marie-Charlotte D.N.J.M. Huysmans^1^

^1^Department of Dentistry, Radboud University Medical Center, Nijmegen, The Netherlands.

^2^Department of Medical Imaging, Radboud University Medical Center, Nijmegen, The Netherlands.

^3^Department of Oral Medicine, Academic Center for Dentistry Amsterdam, University of Amsterdam and VU Amsterdam, Amsterdam, The Netherlands.

^4^Department of Oral and Maxillofacial Surgery, Amsterdam UMC, University of Amsterdam, Amsterdam, The Netherlands.

^5^Department of Oral Medicine/Oral & Maxillofacial Surgery, Atrium Health Carolinas Medical Center, Charlotte, North Carolina, USA.

^6^Department of Otolaryngology/Head and Neck Surgery, Wake Forest University School of Medicine, Winston-Salem, North Carolina, USA.

^7^Department of Oral Microbiology and Immunology, Institute of Odontology, The Sahlgrenska Academy, University of Gothenburg, Gothenburg, Sweden.

^8^Department of Hematology, Radboud University Medical Center, Nijmegen, The Netherlands.

Corresponding author:

Lucky L.A. van Gennip

Department of Dentistry, Radboudumc

Philips van Leydenlaan 25, 6525 EX Nijmegen

The Netherlands

Email address: [Lucky.vanGennip@radboudumc.nl](mailto:Lucky.vanGennip@radboudumc.nl)

# Supplemental material

**Table S1:** Patient-reported gum pain and gum bleeding based on responses to the first two questions of the European Organisation for Research and Treatment of Cancer Quality of Life Questionnaire – Oral Health Module (EORTC QLQ-OH15): ‘”Have you had pain in your gums?" and "Have you had problems with bleeding gums. Responses were recorded using a 4-point Likert scale, with 1 indicating ‘not at all’ and 4 indicating ‘very much’.

|  |  | Baseline  (N = 104) | 3-month  follow-up  (N = 34) | 6-month  follow-up  (N = 45) | 12-month follow-up  (N = 45) | 18-month follow-up  (N = 30) | 5-year  follow-up  (N = 36) |
| --- | --- | --- | --- | --- | --- | --- | --- |
| Gum pain | NMA | 1.2 ±0.4 | 1.1 ±0.4 | 1.1 ±0.3 | 1.5 ±0.6 | 1.2 ±0.4 | 1.1 ±0.4 |
|  | RIC | 1.2 ±0.4 | 1.2 ±0.4 | 1.3 ±0.5 | 1.2 ±0.4 | 1.1 ±0.3 | 1.3 ±0.5 |
|  | MAC | 1.2 ±0.5 | 1.2 ±0.4 | 1.1 ±0.3 | 1.4 ±0.7 | 1.5 ±0.8 | 1.3 ±0.4 |
| Gum bleeding | NMA | 1.1 ±0.3 | 1.1 ±0.3 | 1.1 ±0.2 | 1.3 ±0.5 | 1.3 ±0.5 | 1.4 ±0.5 |
|  | RIC | 1.4 ±0.7 | 1.2 ±0.4 | 1.2 ±0.4 | 1.2 ±0.4 | 1.2 ±0.4 | 1.1 ±0.3 |
|  | MAC | 1.3 ±0.6 | 1.1 ±0.3 | 1.3 ±0.7 | 1.4 ±0.7 | 1.2 ±0.4 | 1.2 ±0.4 |

Values are mean ±SD.
